# LncRNA DLG2-AS1 as a Novel Biomarker in Lung Adenocarcinoma

**DOI:** 10.3390/cancers12082080

**Published:** 2020-07-28

**Authors:** Alberto M. Arenas, Marta Cuadros, Alvaro Andrades, Daniel J. García, Isabel F. Coira, María Isabel Rodríguez, Carlos Baliñas-Gavira, Paola Peinado, Juan Carlos Álvarez-Pérez, Pedro P. Medina

**Affiliations:** 1 Department of Biochemistry and Molecular Biology I, Faculty of Sciences, University of Granada, Av. de Fuente Nueva s/n, 18071 Granada, Spain; alberto.arenas@genyo.es (A.M.A.); alande@ugr.es (A.A.); isabel.fernandezcoira@unige.ch (I.F.C.); carlos.balinas@genyo.es (C.B.-G.); paola.peinado@genyo.es (P.P.); juan.alvarez@genyo.es (J.C.Á.-P.); 2 GENYO, Centre for Genomics and Oncological Research, Pfizer/University of Granada/Andalusian Regional Government, Av. de la Ilustración 114, 18007 Granada, Spain; mcuadros@ugr.es (M.C.); djgargar@ugr.es (D.J.G.); maria.rodriguez@genyo.es (M.I.R.); 3 Department of Biochemistry and Molecular Biology III and Immunology, Faculty of Medicine, University of Granada, Av. de la Investigación 11, 18007 Granada, Spain; 4 Health Research Institute of Granada (ibs.Granada), Av. Fuerzas Armadas 2, 18014 Granada, Spain

**Keywords:** adenocarcinoma of lung, biomarkers, tumor, RNA, long noncoding

## Abstract

Long non-coding RNAs (lncRNAs) are a heterogeneous class of non-coding RNAs whose biological roles are still poorly understood. LncRNAs serve as gene expression regulators, frequently interacting with epigenetic factors to shape the outcomes of crucial biological processes, and playing roles in different pathologies including cancer. Over the last years, growing scientific evidence supports the key role of some lncRNAs in tumor development and proposes them as valuable biomarkers for the clinic. In this study, we aimed to characterize lncRNAs whose expression is altered in tumor samples from patients with lung adenocarcinoma (LUAD) compared to adjacent normal tissue samples. On an RT-qPCR survey of 90 cancer-related lncRNAs, we found one lncRNA, DLG2-AS1, which was consistently downregulated in 70 LUAD patients. To gain insight into its biological function, DLG2-AS1 was cloned and successfully re-expressed in LUAD cancer cell lines. We determined that DLG2-AS1 is not a cis-regulatory element of its overlapping gene DLG2, as their transcription levels were not correlated, nor did DLG2-AS1 restoration modify the expression of DLG2 protein. Furthermore, after generating a receiver operating curve (ROC) and calculating the area under curve (AUC), we found that DLG2-AS1 expression showed high sensitivity and specificity (AUC = 0.726) for the classification of LUAD and normal samples, determining its value as a potential lung cancer biomarker.

## 1. Introduction

Lung cancer is the leading cause of cancer death among both women and men in the US [1]. Non-small cell lung cancer (NSCLC) is the most frequent subtype of lung cancer, accounting for 85% of diagnosed cases [2]. One of the main barriers to further progress in lung cancer therapy is the lack of effective biomarkers for this disease. Thus, research efforts are currently being diverted toward identifying new cancer biomarkers that would improve diagnosis, outcome, and treatment in these cancer patients.

Traditionally, most research about the molecular bases of cancer has focused on finding new oncogenes or tumor suppressor genes within the protein-coding part of the genome. However, recent transcriptomic studies have shown that only 1.22% of the human genome codes for protein, while some transcripts remain as what is called non-coding RNA (ncRNA) [3]. Over the past years, many studies have outlined the important regulatory functions of ncRNAs in the cell, as well as their role in oncogenesis [4]. One example of such ncRNAs are long non-coding RNAs (lncRNAs), which are non-coding transcripts with a span range from 200 nucleotides to 100 kilobases [5].

LncRNAs frequently interface with epigenetic factors to modulate important biological processes such as gene transcription regulation, playing roles in different pathologies including cancer [6]. Thus, some of the biological functions attributed to lncRNAs are related with the expression regulation process, due to their capacity of binding DNA, proteins, and other RNAs [7]. LncRNAs may interact with chromatin remodeling complexes, sequestrate or recruit transcription factors, regulate alternative splicing, serve as competing endogenous RNAs, or control translation at the ribosomes [8]. In addition, lncRNAs can regulate a large fraction of genomic targets working either in trans or in cis, affecting the expression of other genes positively or negatively [9].

Dysregulation of lncRNA expression has been found to be involved in the development of various human cancers. Some lncRNAs have already been validated as oncogenes or tumor suppressor genes in lung cancer. In the case of NSCLC, hundreds of lncRNAs have been associated with tumor development through gene expression microarrays and massive parallel RNA sequencing of lung tumor tissues and paired adjacent non-tumor tissues [10]. However, only a few lncRNAs have been validated with functional assays. For example, MALAT-1 (Metastasis-Associated in Lung Adenocarcinoma Transcript-1) and HOTAIR (HOX Transcript Antisense RNA) have been reported as oncogenic lncRNAs. MALAT-1 expression is upregulated in metastatic NSCLC patients compared to those that do not present metastasis [11]. Furthermore, a novel antisense oligonucleotide (ASO) therapy against MALAT-1 was found to be effective in reducing the extravasation and lung nodule formation capacity of tumor cells both in vivo and in vitro [12]. As another example, HOTAIR expression is described as highly increased in NSCLC compared to normal lung tissues, and it is involved in cell migration, growth, invasion and metastasis, proliferation, and drug resistance [10,13,14,15]. Growing evidence indicates that lncRNAs can also function as tumor suppressor genes, such as TUSC7 (Tumor Suppressor Candidate 7) and LOC285194. TUSC7 is downregulated in NSCLC and its overexpression is shown as a good prognostic biomarker for this disease [16]. LOC285194 expression is decreased in lung cancer tissues and cell lines, and a lower expression of LOC285194 is associated with poor prognosis [17].

Besides, different studies found that lncRNAs appear as a novel and valuable tool for NSCLC prognosis, diagnosis, and treatment. In addition to the aforementioned MALAT-1 and HOTAIR, some lncRNAs have been identified as stable blood biomarkers for NSCLC diagnosis (GAS5, HIF1A-AS1, and XIST); poor prognosis biomarkers (CCAT2 and CARLo-5); and potential targets for molecular therapy (H19) (reviewed by Lu et al. (2018)) [18].

In the present study, we examined the expression levels of 90 lncRNAs in lung adenocarcinoma (LUAD) patient samples and paired adjacent non-tumor tissue. After validating the results, we then focused on DLG2-AS1, our top downregulated lncRNA in tumor tissue. Our functional experiments displayed that DLG2-AS1 is not a cis-regulatory element of DLG2. In addition, after generating a receiver operator curve (ROC) and calculating the area under curve (AUC), we obtained an AUC = 0.726. Thus, our study shows for the first time the downregulation of DLG2-AS1 in LUAD, and its high sensitivity and specificity as a potential novel biomarker for the classification of LUAD and normal samples.

## 2. Results

### 2.1. DLG2-AS1 Expression Is Downregulated in Lung Adenocarcinoma

**Figure 1:**
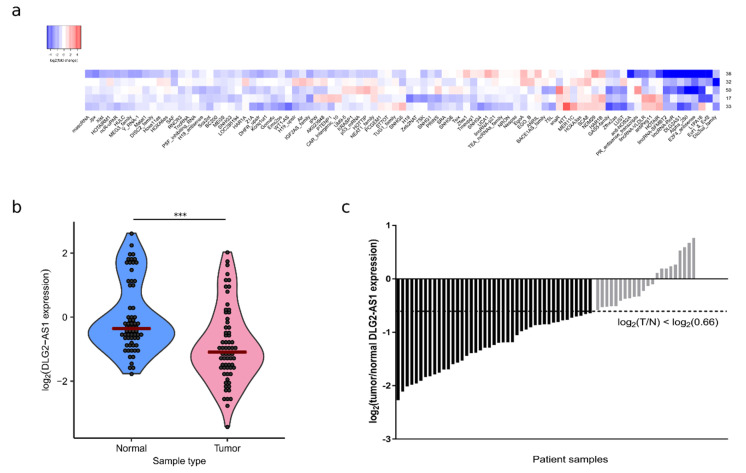
LncRNA DLG2-AS1 is downregulated in lung adenocarcinoma (LUAD). (**a**) heatmap of the 90 lncRNAs analyzed with the Human LncRNA Profile Kit from System Biosciences (SBI); (**b**) DLG2-AS1 expression in normal and tumor samples; and (**c**) tumor/normal fold change (FC(T/N)) of DLG2-AS1 expression in the 65 LUAD patients. Shown in a darker color are the patients who presented DLG2-AS1 downregulation (FC(T/N) < 0.66). *** = *p*-value < 0.001.

To select a dysregulated lncRNA candidate in LUAD patients, we measured the expression of 90 cancer-related lncRNAs in five LUAD tumor samples and adjacent non-tumor tissues (Figure 1a). We found three lncRNAs (DLG2-AS1, E2F4-antisense, and lincRNA-SFMBT2) which were downregulated (tumor/normal fold change < 0.66) in all patient samples.

Next, we measured the expression levels of these three lncRNAs by RT-qPCR using newly-designed oligonucleotides. The lncRNA DLG2-AS1 was the only one whose downregulation could be validated in the initial patients, thus we chose it as the candidate for our study. To extend our results to a broader internal validation set of patients, we measured the expression levels of DLG2-AS1 in 70 LUAD patient samples paired with adjacent non-tumor tissue samples. Five patient samples were discarded due to low quality amplification, possibly because of sample degradation. The expression levels of DLG2-AS1 in tumors were lower than those observed in non-tumor samples (*p* < 0.0001, *n* = 65) (Figure 1b). Out of the 65 analyzed patients, 67.7% (44/65) showed DLG2-AS1 downregulation in the tumor sample (Figure 1c). For the first time, these results show that DLG2-AS1 is downregulated in LUAD patients, suggesting a tumor suppressor role of this lncRNA in LUAD.

### 2.2. DLG2-AS1 Is Not a Cis-regulator of DLG2 Expression

Due to previous evidence showing that some antisense lncRNAs may exert their function in cis by up- or downregulating their overlapping protein-coding genes [19], we checked if there was any correlation between the expression of DLG2-AS1 and the expression of its overlapping protein-coding gene, DLG2.

First, we studied the expression levels of DLG2-AS1 and DLG2 in a set of 20 matched tumor-normal patients, finding no significant correlation (Pearson correlation *r* = 0.378, *p* = 0.1, *n* = 20). To corroborate this result, we also analyzed an external dataset of 12 tumor-normal paired samples from LUAD available at TCGA data portal (https://portal.gdc.cancer.gov). Analysis of this external dataset confirmed the lack of correlation between DLG2-AS1 and DLG2 in patient samples (Pearson correlation *r* = 0.16, *p* = 0.62, *n* = 12).

**Figure A1:**
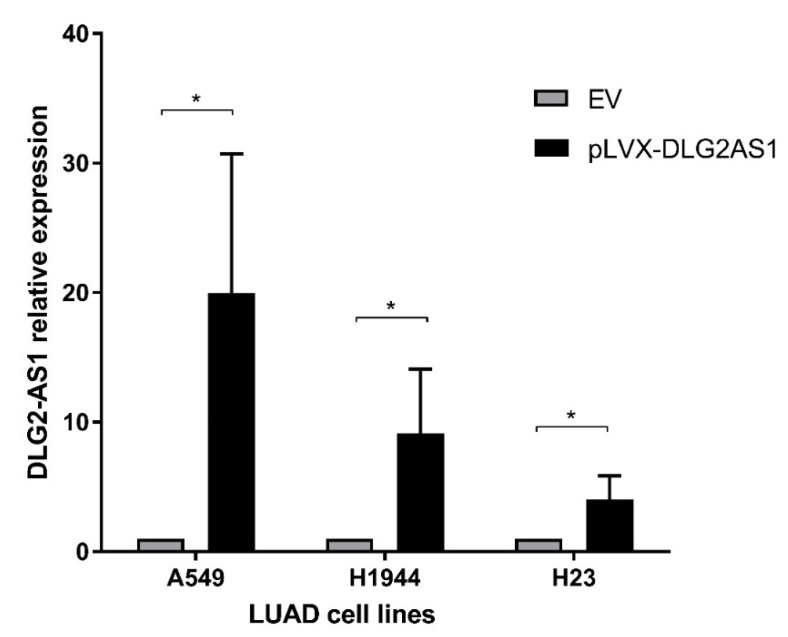
DLG2-AS1 restoration model on lung adenocarcinoma (LUAD) cell lines successfully overexpresses DLG2-AS1. DLG2-AS1 relative expression was measured by RT-qPCR 5 days after transfection, and normalized by the expression of the reference gene U1 snRNA. In all three cell lines tested, DLG2-AS1 was successfully overexpressed (relative expression (pLVX-DLG2AS/EV) > 2). * = *p*-value < 0.05.

**Figure A2:**
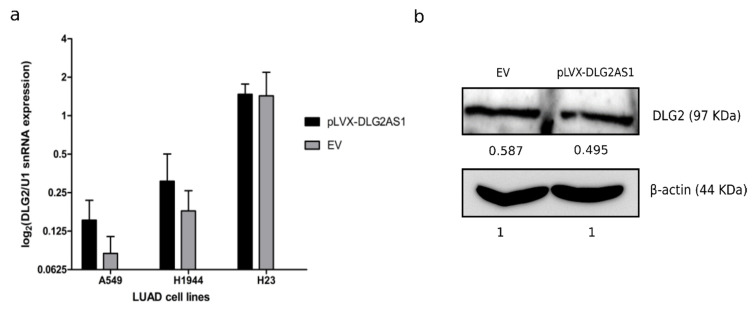
DLG2-AS1 is not a cis-regulator of DLG2 expression. (**a**) RNA expression levels of DLG2 were unaffected after DLG2-AS1 overexpression (pLVX-DLG2AS1) compared to empty vector transfection (EV). Results from RT-qPCR were normalized to expression of the reference gene U1 snRNA. (**b**) Protein levels of human DLG2 did not change upon transfection with pLVX-DLG2AS1 compared to EV in two independent Western blot experiments (relative pLVX-DLG2AS1/EV densitometry measure mean = 0.984 ± 0.199, *p* = 0.929). Uncropped WB images are available at the Supplementary Material zip file.

Furthermore, we generated a restoration model of DLG2-AS1 using a plasmid construction (pLVX-DLG2AS1-IRES-zsGreen1). The restoration model was successful, as DLG2-AS1 expression levels were restored by transient transfection in three LUAD cell lines (A549, H1944, and H23) even at five days after transfection (Figure A1). Then, we measured DLG2 expression in these cell lines and we observed that the levels of DLG2 were unaffected by DLG2-AS1 overexpression (Figure A2a).

Finally, we considered that some lncRNAs may also regulate their neighboring genes at a translational level by interacting with the ribosome [20]. Such lncRNAs might not affect the mRNA expression levels of the genes they are regulating, but their protein expression levels. To fully discard this option, we measured the protein expression levels of DLG2 by Western blot after introducing a plasmid containing DLG2-AS1 (pLVX-DLG2AS1) or the empty vector (EV). However, we observed no significant differences in DLG2 protein levels between pLVX-DLG2AS1 and EV-transfected cells (Figure A2b). Therefore, we ruled out DLG2-AS1 as cis-regulator of DLG2 expression in LUAD patient samples.

### 2.3. LncRNA DLG2-AS1 Expression Shows Potential as a Lung Adenocarcinoma Biomarker

**Figure 2:**
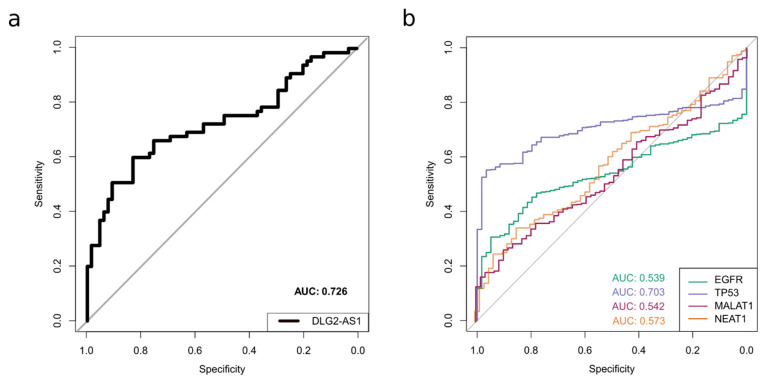
DLG2-AS1 shows potential as a lung adenocarcinoma (LUAD) biomarker: (**a**) receiver operating characteristic (ROC) curve and area under curve (AUC) value of DLG2-AS1 expression in our cohort of LUAD patients; and (**b**) ROC curves and AUC values of other LUAD biomarkers (EGFR, TP53, MALAT-1, and NEAT1), obtained from TCGA data.

Next, we assessed the usefulness of DLG2-AS1 expression as a biomarker for the classification of samples as tumor or normal. Using the data from our patient cohort (*n* = 65 tumor-normal pairs), a receiver operating characteristic (ROC) curve was generated. For each possible threshold value of DLG2-AS1 expression, samples were classified as tumor or normal based on whether DLG2-AS1 expression was below or above the threshold. Sensitivity and specificity of the classification were assessed for each possible threshold value, and they were plotted yielding the ROC curve. Then, the area under curve (AUC) was calculated as a measure of the idoneity of DLG2-AS1 expression to distinguish between tumor and normal patients. The AUC of DLG2-AS1 was 0.726 (95% CI = 0.638–0.815) showing a threshold of −0.916 for log2 (Delta-Cq), an optimal specificity of 80%, and a sensitivity of 60% (Figure 2a). This suggests that DLG2-AS1 expression may be useful as a tumor biomarker.

To compare its specificity and sensitivity with other well-known cancer biomarkers [21] and cancer-related lncRNAs [18], we calculated the ROC curves of the EGFR, TP53, and TP53 genes, as well as the oncogenic lncRNAs MALAT-1 and NEAT1, using TCGA-LUAD data. We obtained AUC values of 0.539, 0.703, 0.542, and 0.573, respectively, which are considerably lower than the AUC values obtained for DLG2-AS1 (Figure 2b).

## 3. Discussion

Despite diagnostic and therapeutic advances, lung cancer remains highly lethal, and the five-year relative survival rate remains at 19%, meaning that four out of five lung cancer patients will perish within five years after being diagnosed [1]. Standard therapeutic strategies, such as surgery, chemotherapy, or radiotherapy, appear to have reached a plateau [22]. Although genetic alterations have been associated with the development and progression of lung cancer, the underlying molecular mechanisms remain unclear. Therefore, a greater understanding of the biology of this disease is urgently required to support the development of novel therapeutic approaches.

Multiple lines of evidence increasingly link dysregulations of lncRNAs to diverse human diseases including lung cancer. Recent scientific progress suggests that some lncRNAs could have a significant role in diagnosis, prognosis, and treatment of human diseases [18]. In fact, several studies have proven the value of lncRNAs as cancer biomarkers in the clinic. For example, the lncRNA PCA3 was found to be useful for the early diagnosis of prostate cancer. In this way, the U.S. Food and Drug Administration (FDA) approved the clinical use of a PCA3 detection kit for patients under suspicion of requiring a biopsy for the diagnosis of prostate cancer in 2012 [23]. Therefore, the identification and research of cancer-associated lncRNAs is critical for understanding the roles of lncRNAs in the carcinogenesis and improving the current clinic.

DLG2-AS1, also known as AP001825.2 (NCBI Gene ID: 100302690), is an antisense lncRNA within the first intron of the overlapping protein-coding gene DLG2 (Disks Large Homolog 2). DLG2-AS1 and DLG2 are mainly expressed in brain adult tissues, as DLG2 is a MAGUK family protein for which a role in NMDA-receptor assembly has been proposed [24]. DLG2 is a homologous protein to Drosophila’s dlg-A, which is considered a tumor suppressor protein [25]. In humans, a DLG2 isoform was overexpressed in renal oncocitoma [26]. Regarding DLG2-AS1 expression, Polesskaya et al. (2003) showed a downregulation in brain tissues from patients with schizophrenia [27]. However, before our study, no information of DLG2-AS1/DLG2 in lung cancer was published. Thus, for the first time, we detected a DLG2-AS1 downregulation in LUAD patients.

Gene expression regulation roles of lncRNAs depend on their molecular way of action, including interfering with transcription, messenger RNA (mRNA) maturation, and mRNA stability or translation [8]. LncRNAs may act regulating in cis the expression of neighboring genes, or in trans modulating distant gene expression. Antisense lncRNAs such as DLG2-AS1 are transcribed from the opposite strand of other genes, and they usually regulate in cis the expression of their overlapping protein-coding genes [19]. However, according to our results, there is no correlation on the expression of DLG2-AS1 and DLG2, neither in patient samples nor in our DLG2-AS1 restoration cell models. Thus, our results do not support the modulating role in cis of DLG2-AS1 over DLG2 expression. Further regulating roles in trans could be discovered in future mechanistic studies, as was done with other trans-acting lncRNAs such as HOTAIR [28], NEAT1 [29], MEG1 [30], and Bvht [31,32].

Finally, we were interested in determining whether DLG2-AS1 can serve as a good diagnostic biomarker for LUAD patients. The ROC curves that we conducted to analyze the diagnostic power of DLG2-AS1 showed that DLG2-AS1 has a relatively high diagnostic value for the LUAD patients in comparison with other broadly studied and validated LUAD biomarkers (EGFR, TP53), as well as other lncRNAs with proven clinical value (MALAT-1, NEAT1). These results suggest a potential use of DLG2-AS1 as a LUAD biomarker for the diagnosis of the disease. Nevertheless, further analyses, such as its detection in exosomes or by liquid biopsy, would be necessary to completely validate its clinical use as a LUAD biomarker.

## 4. Materials and Methods

### 4.1. Sample Collection and Ethics Approval

RNA samples from lung adenocarcinoma (LUAD) patients were obtained from the Basque Biobank (Bilbao, Spain). Participants provided written consent in accordance to the procedures of the Declaration of Helsinki and the institutional and national guidelines. Seventy tumor samples were taken from primary malignant LUAD tumors, as well as their adjacent non-tumor tissues, based on macroscopic examination by trained pathologists. The study was approved by the Ethics Committee (CEI Granada, Spain), Department of Health, Andalusian Regional Government, and by the Basque Foundation for Health Innovation and Research, Spain (CES-BIOEF 2015-18).

### 4.2. LncRNA Profiling and Candidate Selection

LncRNA expression profiles were studied in LUAD patient samples paired with their adjacent non-tumor samples by reverse transcription-quantitative polymerase chain reaction (RT-qPCR), using the Human LncRNA Profiler kit (System Biosciences (SBI), Palo Alto, CA, USA). For the RT-qPCR validation, RNA was reverse-transcribed to copy DNA (cDNA) using a RevertAid RT kit (ThermoScientific, Waltham, MA, USA). All qPCRs were performed using the KAPA SYBR^®^ FAST qPCR Master Mix (Merck KGaA, Darmstadt, Germany). Reactions were set up following the manufacturers’ recommendations. Relative expression was calculated using the Delta-Cq method and normalizing by the expression of the reference gene U1 snRNA. The sequences of used oligonucleotides are shown in Appendix A, Table A1.

### 4.3. Cell Culture

The A549, H1944, and H23 LUAD cell lines were purchased from the American Type Culture Collection (ATCC), and they were kept in culture in T-75 culture flasks for a maximum time of two months, changing the medium and splitting the cells every 3–5 days depending on their confluence state. DMEM High Glucose medium (Biowest, Nuaillé, France) supplemented with 10% fetal bovine serum (FBS) and 1% penicillin/streptomycin/amphotericin (P/S/A) was used for the A549 cell line. RPMI 1640 medium (Biowest) supplemented with 10% FBS, 1% P/S/A and 1% L-glutamine was used for the H1944 and H23 cell lines.

### 4.4. Cloning of DLG2-AS1 Vector and Transfection

The empty vector (EV) pLVX-IRES-zsGreen1 (Clontech, Mountain View, CA, USA) was used to introduce the lncRNA DLG2-AS1 into the cells. Two oligonucleotides containing overhanging restriction sites for EcoRI and XbaI were designed (sequences shown in Appendix A, Table A1). To amplify the insert, a PCR was performed using Phusion High-Fidelity DNA Polymerase (ThermoScientific). The ~200 bp insert was purified from an 1.5% agarose gel and then double-digested along with the EV using enzymes EcoRI and XbaI (ThermoScientific). The cut insert and EV were ligated using T4 ligase (New England Biolabs (NEB), Ipswich, MA, USA) and then the resulting plasmid was introduced into chemically competent bacteria for its amplification. All reactions were set up following the manufacturers’ recommendations.

For the transfection, A549, H1944, and H23 cells were plated in six-well plates and cultured until a cell confluence of 60–70% was reached. Transfection with a plasmid containing the DLG2-AS1 sequence (pLVX-DLG2AS1) or the empty vector (EV) was performed using TransIT reagent (Mirus Bio, Madison, WI, USA) according to the manufacturer’s recommended reaction settings. Cells were incubated in OptiMEM medium (ThermoScientific) with the plasmid and the TransIT reagent for 24 h and then transfection medium was replaced by fresh medium. Overexpression of DLG2-AS1 was then checked by RT-qPCR.

### 4.5. DLG2 Protein Quantification

Total protein was extracted from cellular pellets and then a Western blot was performed with 100 μg of protein in a 10% acrylamide gel. Human DLG2 protein was detected using the anti-PSD-93 antibody (CAT# APZ-002, Alomone Labs, Jerusalem, Israel). Human BETA-ACTIN was used as a loading control protein and detected using the anti-β-actin monoclonal antibody (CAT# A5441, Sigma-Aldrich, St. Louis, MO, USA). Western blot bands were digitalized and then analyzed by densitometry using the ImageJ software.

### 4.6. Analysis of TCGA-LUAD Data

We downloaded raw RNA-Sequencing (RNA-Seq) gene expression counts from the TCGA-LUAD project (*n* = 533) using the Genomic Data Commons Data Transfer Tool (v 1.4.0) (9 May 2019). We analyzed the RNA-Seq data using the R package “edgeR” (R version 3.5.3, Bioconductor version 3.8). We normalized the data using the trimmed mean of M-values method and extracted the counts per million (CPM) for DLG2-AS1 (ENSG00000274006.1) and DLG2 (ENSG00000150672.15).

### 4.7. Statistical Analysis

Two-tailed Student’s *t*-test considering mean and standard deviation was used to determine whether the difference between two sets of normally-distributed data is significant. Correlation between the expression of DLG2 and DLG2-AS1 was calculated using Pearson’s correlation test. For the ROC curves, we fitted logistic regression models to predict the classification of samples as “tumor” or “normal” based on gene expression.

## 5. Conclusions

We determined that DLG2-AS1 is a lung cancer biomarker with high sensitivity and specificity (AUC = 0.726) for the classification of LUAD and normal samples. Additionally, we concluded that DLG2-AS1 is not a cis-regulatory element of its overlapping gene DLG2, as their transcription levels were not correlated, nor did DLG2-AS1 restoration modify the expression of DLG2 protein in our lung cancer cellular models.

## Supplementary Materials

The following are available online at https://www.mdpi.com/2072-6694/12/8/2080/s1, File 1: Uncropped Western blot images.

## Appendix A

**Table A1:** Sequences of used oligonucleotides for qPCR and vector cloning.

Oligonucleotide Name	5′ ➔ 3′ Sequence
DLG2-AS1 Fw	ATCCGGATGTGAGGTTATAAT
DLG2-AS1 Rv	AATCCAGATCCCAAGACTTC
DLG2 Fw	GGGAGATACCATGATCACG
DLG2 Rv	CCACAAATTATGCAGTCGAGTTTCCC
U1 snRNA Fw	GAAGACCTCATTCTTTCCTATG
U1 snRNA Rv	CGGCTTCTATAAACTTGTGC
AAAA-EcoRI-DLG2-AS1	AAAAGAATTCTTAACTTGTTAATCA
TTTT-XbaI-DLG2-AS1	TTTTTCTAGACCAGATGGTCAGTGA
