# Supplementary material for: LncRNA DLG2-AS1 as a Novel Biomarker in Lung Adenocarcinoma
Source: Cancers (Basel). 2020 Jul 28;12(8):2080. doi: 10.3390/cancers12082080 (PMC7463504; doi:10.3390/cancers12082080)
Supplement: Supplementary file 1 [file cancers-12-02080-s001.zip › WB uncropped images and lanes.pptx]

## Slide 1
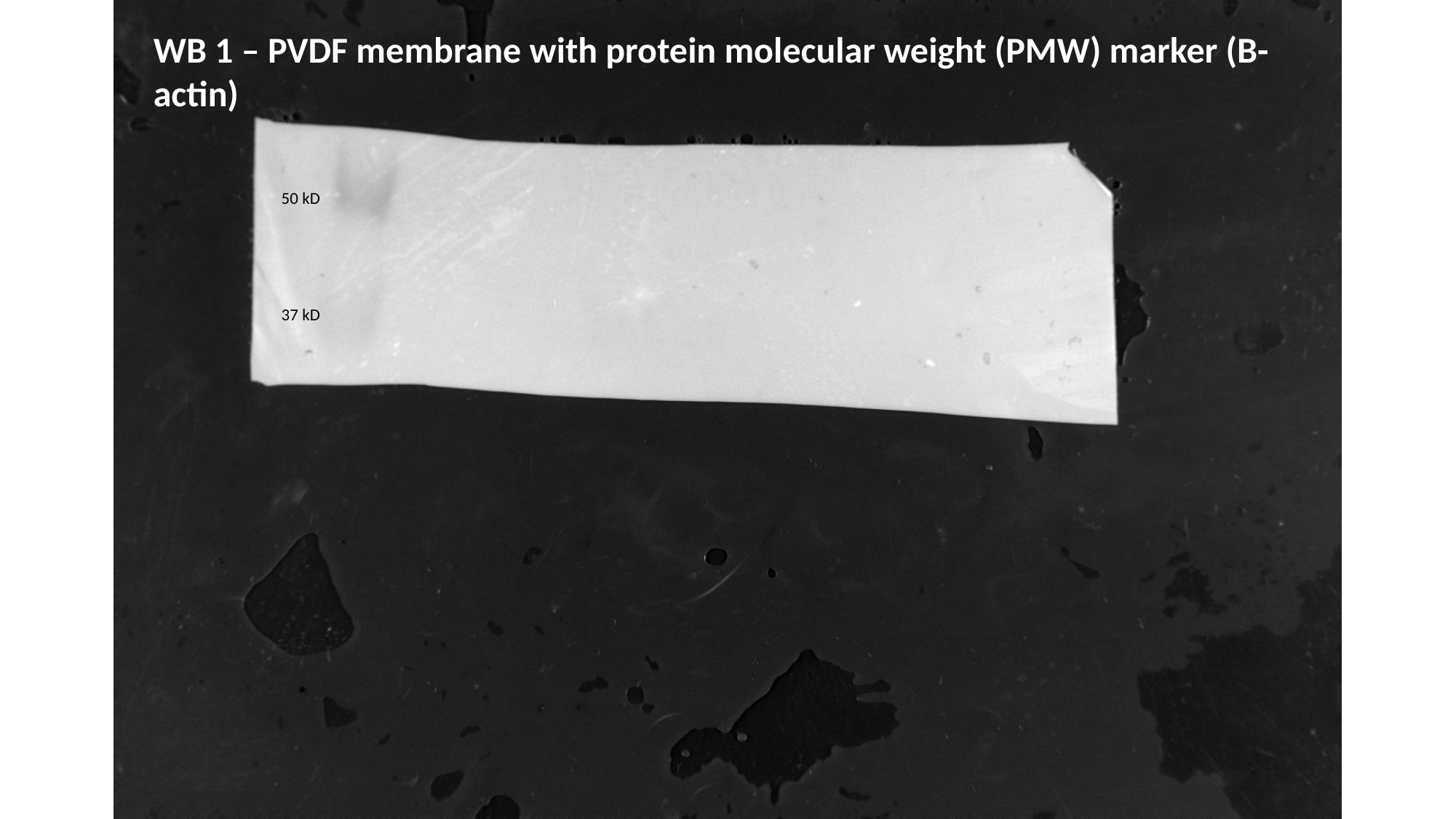

WB 1 – PVDF membrane with protein molecular weight (PMW) marker (B-actin)
50 kD
37 kD

## Slide 2
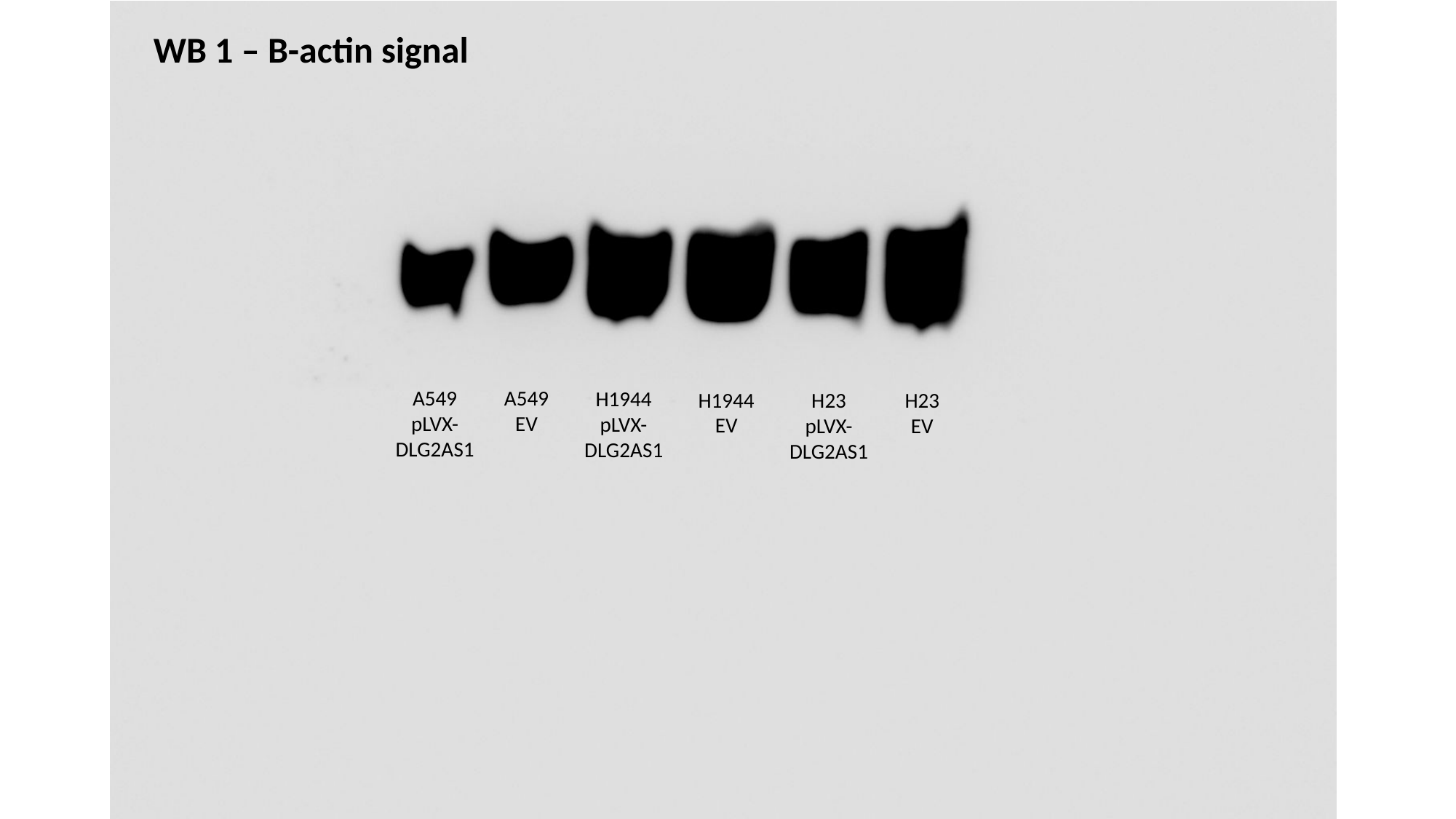

WB 1 – B-actin signal
A549
pLVX-DLG2AS1
A549
EV
H1944
pLVX-DLG2AS1
H1944
EV
H23
pLVX-
DLG2AS1
H23
EV

## Slide 3
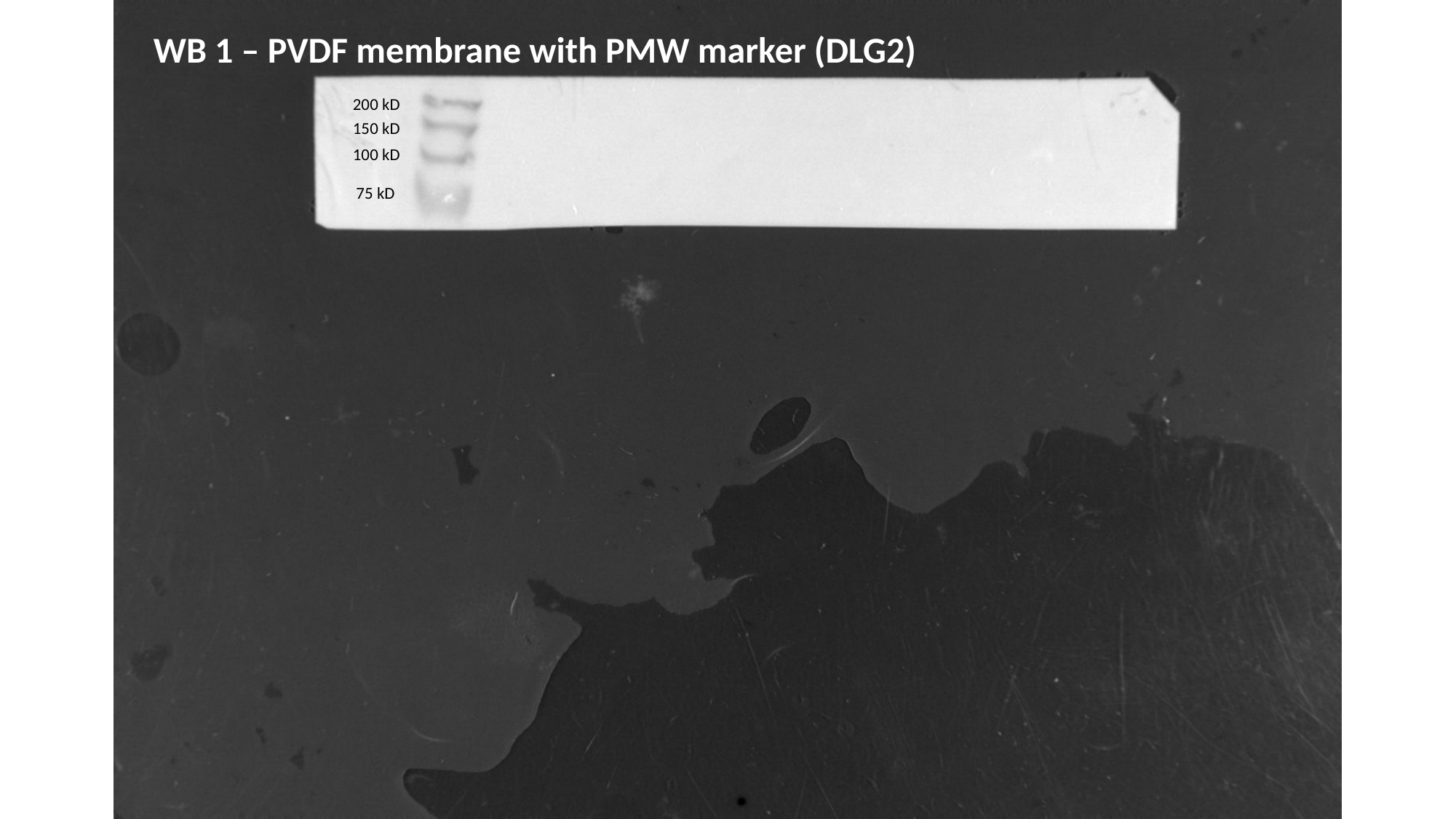

WB 1 – PVDF membrane with PMW marker (DLG2)
200 kD
150 kD
100 kD
75 kD

## Slide 4
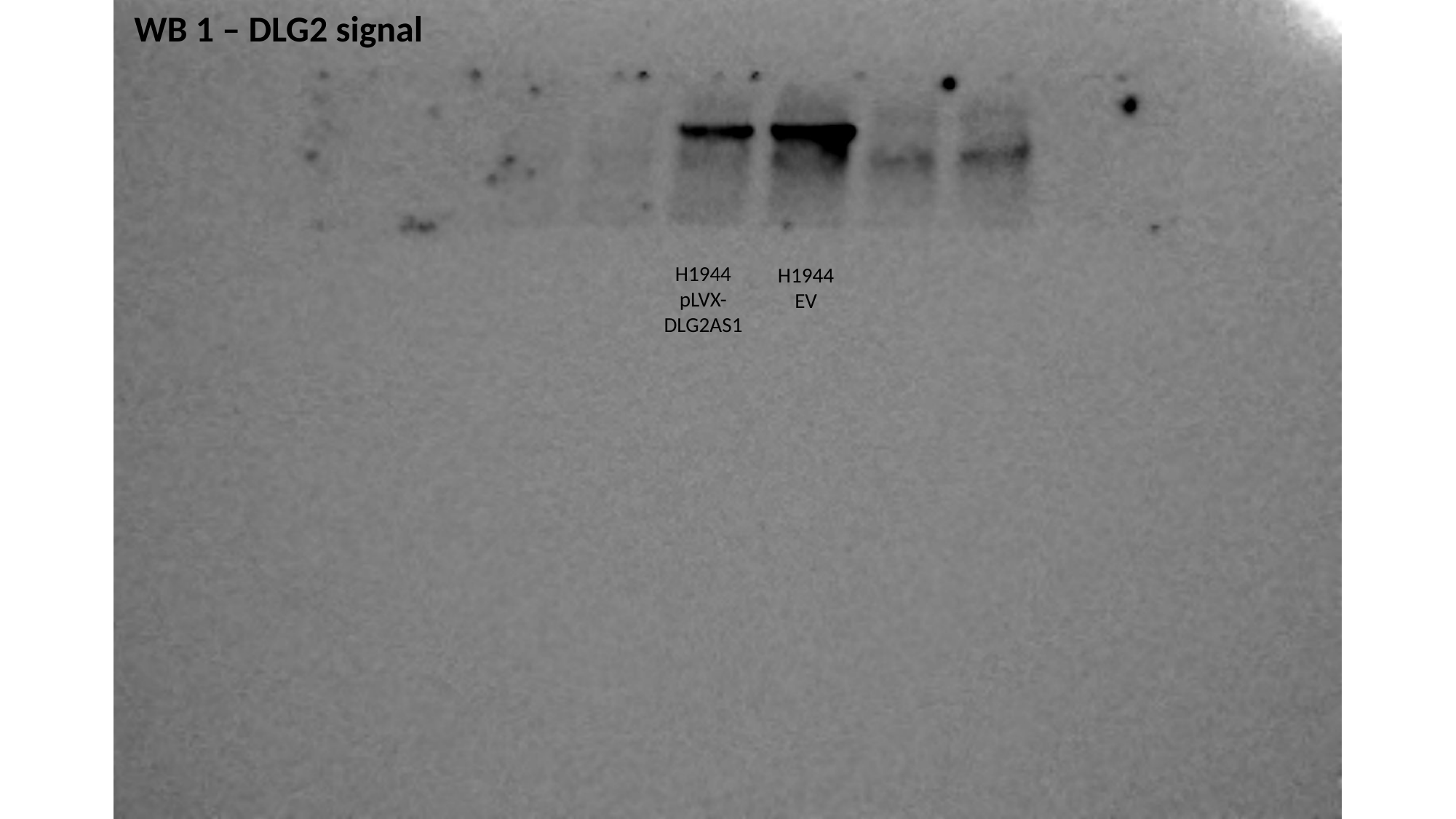

WB 1 – DLG2 signal
H1944
pLVX-DLG2AS1
H1944
EV

## Slide 5
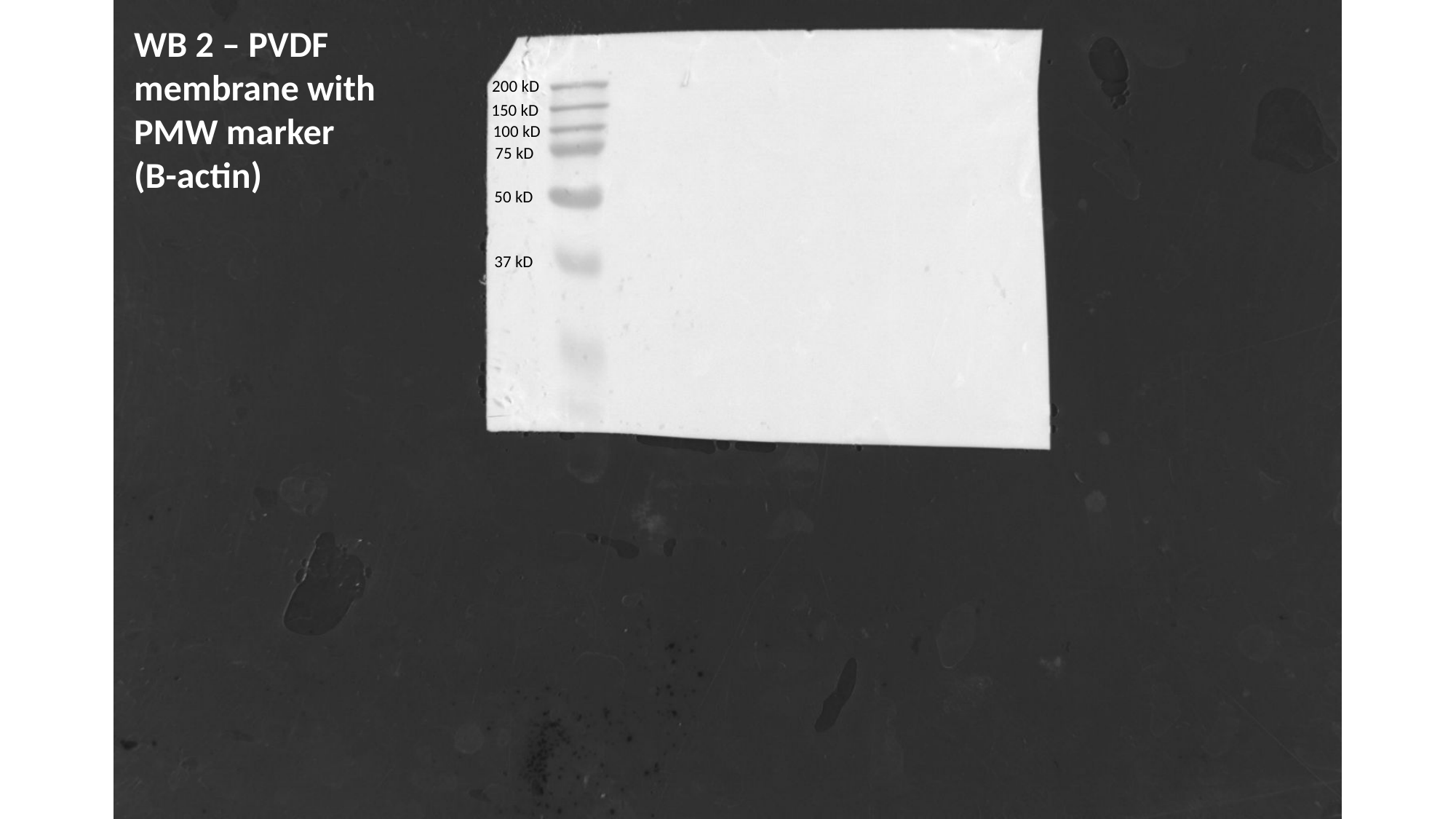

WB 2 – PVDF membrane with PMW marker
(B-actin)
200 kD
150 kD
100 kD
75 kD
50 kD
37 kD

## Slide 6
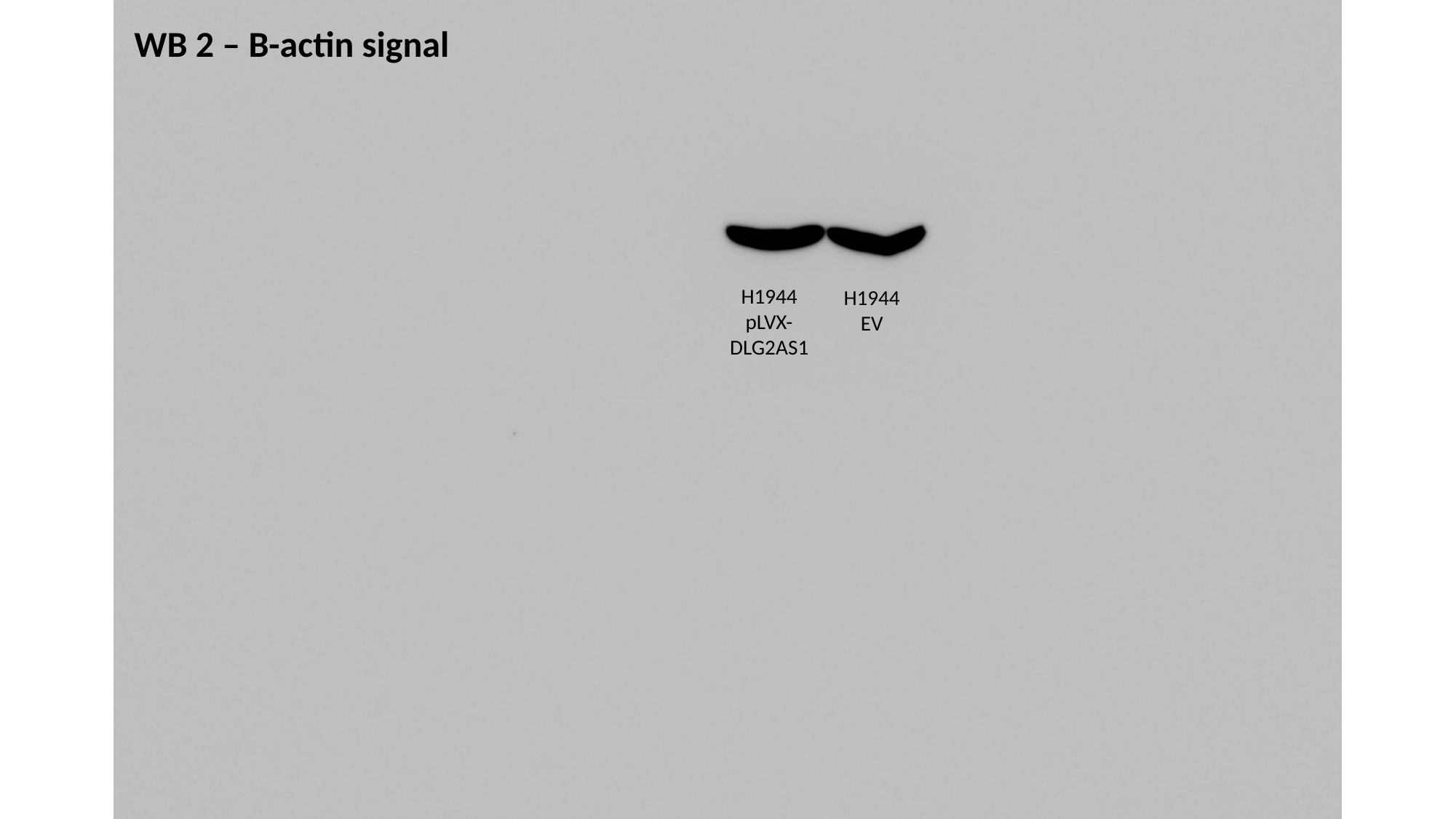

WB 2 – B-actin signal
H1944
pLVX-DLG2AS1
H1944
EV

## Slide 7
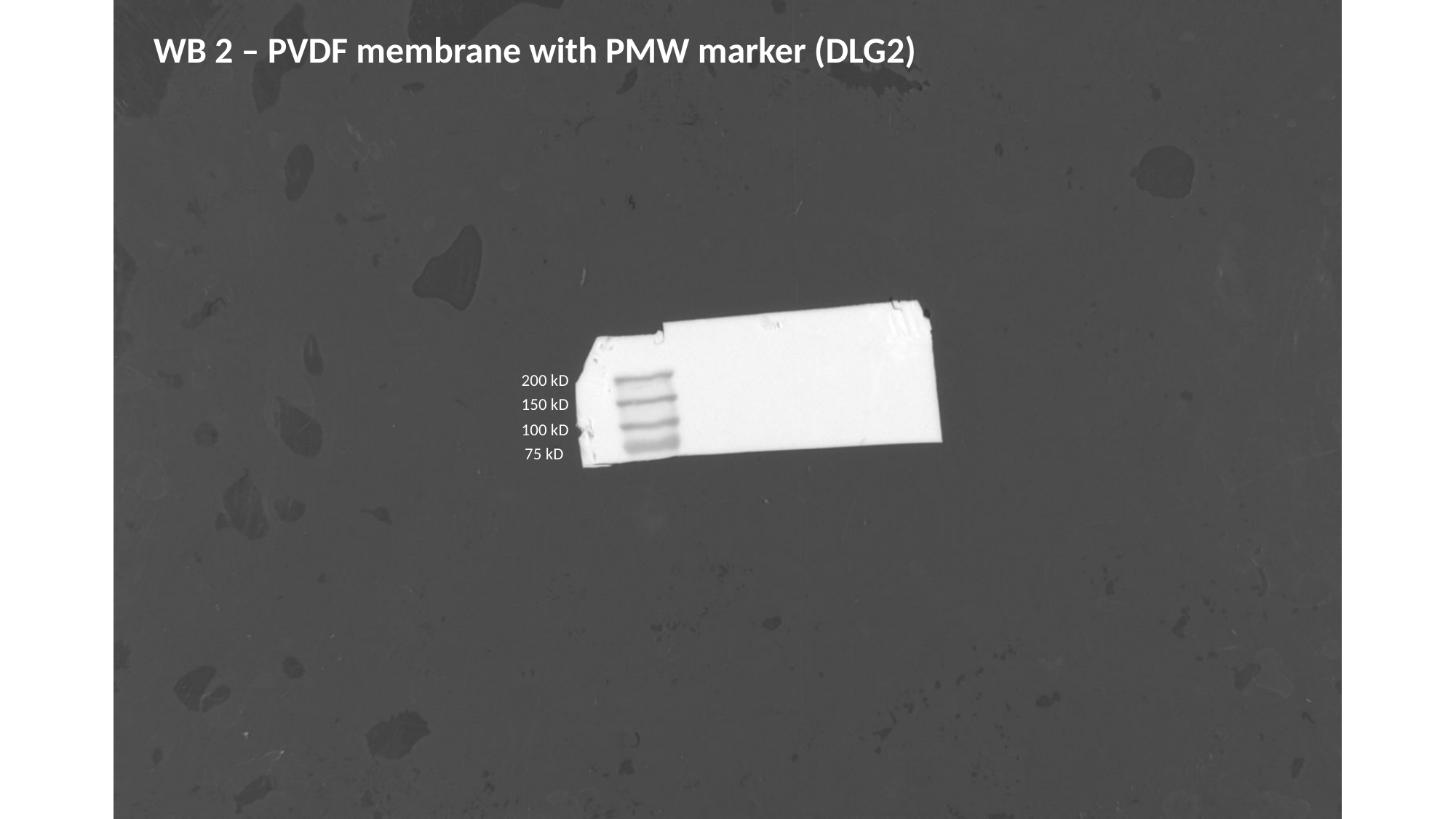

WB 2 – PVDF membrane with PMW marker (DLG2)
200 kD
150 kD
100 kD
75 kD

## Slide 8
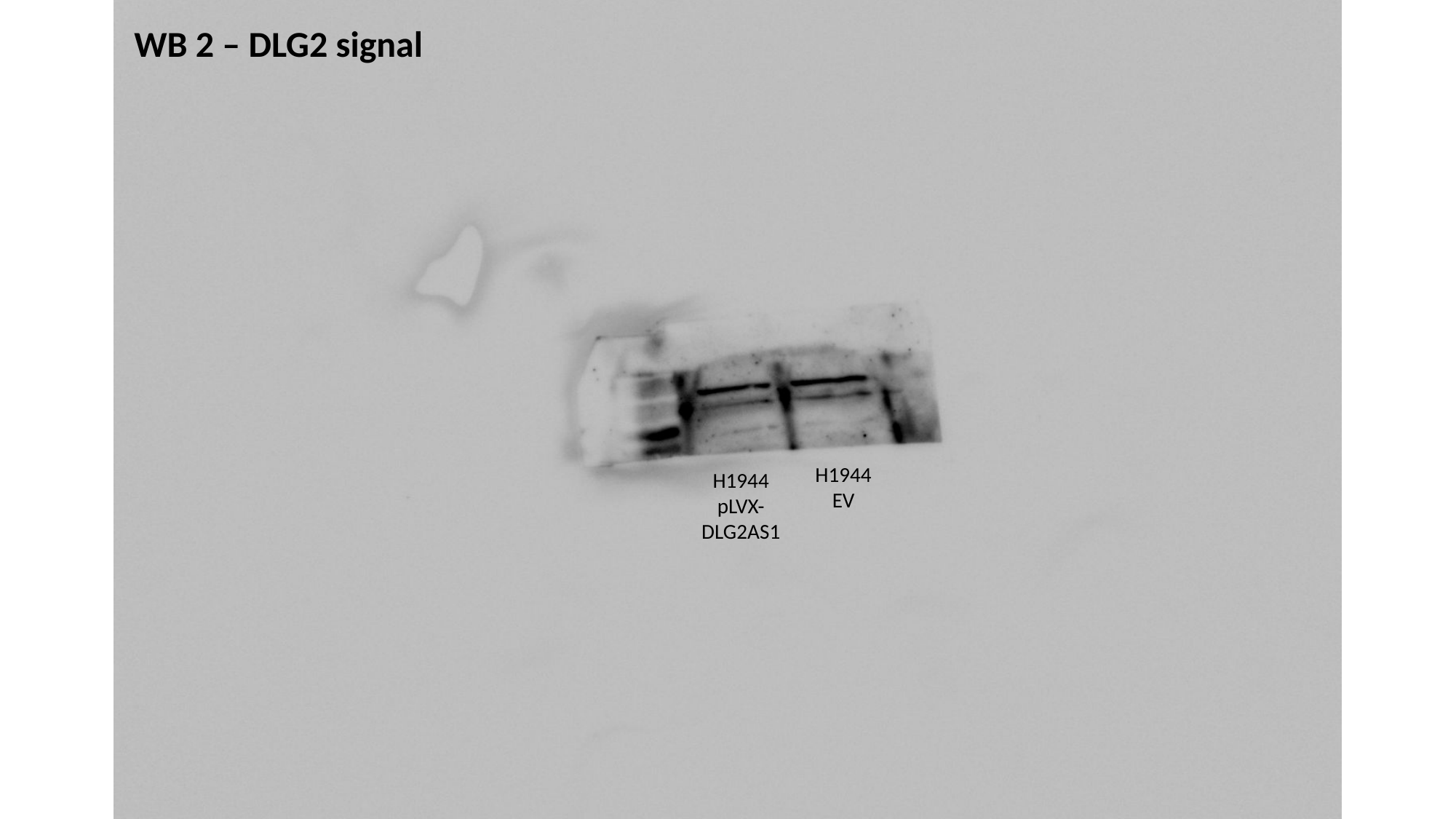

WB 2 – DLG2 signal
H1944
EV
H1944
pLVX-DLG2AS1
